# Supplementary material for: Implementation of a Model-Based Programme to Promote Personal and Social Responsibility and Its Effects on Motivation, Prosocial Behaviours, Violence and Classroom Climate in Primary and Secondary Education
Source: Int J Environ Res Public Health. 2019 Nov 2;16(21):4259. doi: 10.3390/ijerph16214259 (PMC6862051; doi:10.3390/ijerph16214259)
Supplement: Supplementary file 1 [file ijerph-16-04259-s001.pdf]

CUESTIONARIO DE RESPONSABILIDAD ESCOLAR

Esta encuesta forma parte de un estudio de la Facultad de Ciencias del Deporte de la Universidad de Murcia.

Por favor, dedícanos parte de tu tiempo para contestar a una serie de preguntas acerca del tema que nos ocupa.

En todo momento estos datos se tratarán de forma anónima; tus respuestas son totalmente confidenciales.

INSTRUCCIONES

- Marca con una X la respuesta que tú consideres adecuada y completa las preguntas con bolígrafo si es posible.
- En caso de equivocación, rellena completamente y marca de nuevo con una X sobre la respuesta que creas correcta. **No poner respuestas dobles**
- **CÓDIGO IDENTIFICADOR** _______________
- **Género**: □ Masculino (chico). □ Femenino (chica). **Fecha de nacimiento**: ____/____/_____

¿Qué quieres hacer en el futuro a nivel educativo?:

□ Hacer Bachillerato o Formación Profesional Media. □ Ir a Universidad o Formación Profesional (FP) Superior.
□ Terminar ESO o FP Básica y empezar a Trabajar. □ Dejar los estudios antes de hacer la ESO o FP Básica.

Las siguientes preguntas buscan ver los motivos que tienes para ir al colegio. Contesta con sinceridad siguiendo la escala que te presentamos a continuación:

No se corresponde en absoluto	Se corresponde muy poco	Se corresponde poco	Se corresponde medianamente	Se corresponde mucho	Se corresponde casi totalmente	Se corresponde totalmente
1	2	3	4	5	6	7

Voy al colegio…	1	2	3	4	5	6	7
1. Porque necesito, al menos, un título para encontrar un trabajo bien pagado.							
2. Porque siento placer y satisfacción cuando aprendo nuevas cosas.							
3. Porque creo que haber cursado primaria me ayudará a prepararme mejor para la profesión que elegiré.							
4. Porque realmente me gusta asistir a clase.							
5. Sinceramente no lo sé, creo que estoy perdiendo el tiempo en el colegio.							
6. Por el placer que siento cuando me supero en los estudios.							
7. Para demostrarme que soy capaz de terminar primaria.							
8. Para conseguir un puesto de trabajo más prestigioso.							
9. Por el placer que siento cuando descubro cosas nuevas que nunca había visto antes.							
10. Porque me permitiría acceder al mercado laboral en el campo que más me gusta.							
11. Porque para mí, el colegio es divertido.							
12. Antes tenía buenas razones para ir al colegio, pero ahora me pregunto si vale la pena continuar.							
13. Por el placer que siento cuando consigo uno de mis objetivos personales.							
14. Porque cuando hago bien las tareas en clase me siento importante.							
15. Porque quiero "vivir bien" una vez que termine mis estudios.							
16. Por el placer que siento al ampliar mis conocimientos sobre los temas que me interesa.							
17. Porque me ayudará a tomar una mejor decisión en lo que respecta a mi orientación profesional.							
18. Por el placer que siento cuando participo en debates con profesores interesantes.							
19. No sé por qué voy al colegio y, sinceramente, no me importa.							
20. Por la satisfacción que siento cuando voy superando actividades académicas difíciles.							
21. Para demostrarme que soy una persona inteligente.							
22. Para poder conseguir, posteriormente un mejor salario.							
23. Porque mis estudios me permiten seguir aprendiendo muchas cosas que me interesan.							
24. Porque creo que la educación que recibo en el colegio mejorará mi competencia laboral.							
25. Porque me estimula leer sobre los temas que me interesan.							
26. No lo sé, no entiendo que hago en el colegio.							
27. Porque las clases me producen satisfacción personal cuando trato de conseguir lo máximo en mis estudios.							
28. Porque quiero demostrarme que puedo superar mis estudios.							

Las siguientes preguntas hacen referencia a cómo te sientes en las clases. Contesta siguiendo la escala siguiente:

Totalmente en desacuerdo	Algo en desacuerdo	Neutro	Algo de acuerdo	Totalmente de acuerdo
1	2	3	4	5

En mis clases…	1	2	3	4	5
1. Las actividades que realizo se ajustan a mis intereses.					
2. Siento que he tenido una gran progresión con respecto al objetivo final que me he propuesto.					
3. Me siento muy cómodo/a cuando hago actividades de clase con los demás compañeros/as.					
4. La forma de realizar las actividades coincide perfectamente con la forma en que yo quiero hacerlos.					
5. Realizo las actividades de clase eficazmente.					
6. Me relaciono de forma muy amistosa con el resto de mis compañeros/as.					
7. La forma de realizar las actividades de clase responde a mis deseos.					
8. Las actividades de clase son algo que hago muy bien.					
9. Siento que me puedo comunicar abiertamente con mis compañeros/as.					
10. Tengo la oportunidad de elegir como realizar las actividades de clase.					
11. Pienso que puedo cumplir con las exigencias de la clase.					
12. Me siento muy cómodo con los compañeros/as de clase.					

Indica ahora **tu grado de acuerdo o desacuerdo con las afirmaciones** que vienen a continuación.

	1	2	3	4	5
1. Cuando hay una emergencia, hay alguien para ayudarme.					
2. Los profesores de este centro son agradables con los estudiantes.					
3. Trabajo en los deberes escolares.					
4. Cuando los estudiantes rompen las reglas son tratados justamente.					
5. El colegio está muy ordenado y limpio.					
6. Se puede confiar en la mayoría de la gente de este colegio.					
7. Los estudiantes realmente quieren aprender.					
8. Los profesores me dicen cuando hago un buen trabajo.					
9. Los estudiantes de todas las razas y grupos étnicos son respetados.					
10. Mi clase tiene un aspecto muy agradable.					
11. La gente de este colegio se cuida uno al otro.					
12. Mi colegio es un lugar muy seguro.					
13. Los profesores hacen un buen trabajo buscando a los alborotadores.					
14. Me siento cómodo hablando con mis profesores de mis problemas.					

Lo normal es comportarse unas veces bien y otras mal. Nos interesa saber **cómo te comportas normalmente durante las clases**. No hay respuestas correctas o incorrectas. Por favor, responde siguiendo la siguiente escala:

Totalmente en desacuerdo	Bastante en desacuerdo	Algo en desacuerdo	Algo de acuerdo	Bastante de acuerdo	Totalmente de acuerdo
1	2	3	4	5	6

En mis clases…	1	2	3	4	5	6
1. Respeto a los demás.						
2. Respeto a mi profesor.						
3. Ayudo a otros.						
4. Animo a los demás.						
5. Soy amable con los demás.						
6. Controlo mi temperamento.						
7. Colaboro con los demás.						
8. Participo en todas las actividades.						
9. Me esfuerzo.						
10. Me propongo metas.						
11. Trato de esforzarme, aunque no me guste la tarea.						
12. Quiero mejorar.						
13. Me esfuerzo mucho.						
14. No me propongo ninguna meta.						

CUESTIONARIO DE RESPONSABILIDAD ESCOLAR

Esta encuesta forma parte de un estudio de la Facultad de Ciencias del Deporte de la Universidad de Murcia.

Por favor, dedícanos parte de tu tiempo para contestar a una serie de preguntas acerca del tema que nos ocupa.

En todo momento estos datos se tratarán de forma anónima; tus respuestas son totalmente confidenciales.

INSTRUCCIONES

- Marca con una X la respuesta que tú consideres adecuada y completa las preguntas con bolígrafo si es posible.
- En caso de equivocación, rellena completamente y marca de nuevo con una X sobre la respuesta que creas correcta. **No poner respuestas dobles**
- **CÓDIGO IDENTIFICADOR** _______________
- **Género:** □ Masculino (chico). □ Femenino (chica). **Fecha de nacimiento:** ____/____/_____

Las siguientes preguntas buscan ver **como percibes los actos de violencia bajo tu punto de vista** en tu centro, contesta a las frases reflexionando brevemente sobre cada una de ellas. Contesta siguiendo la escala siguiente:

Totalmente en desacuerdo	Algo en desacuerdo	Neutro	Algo de acuerdo	Totalmente de acuerdo
1	2	3	4	5

	1	2	3	4	5
1. El alumnado pone motes molestos a sus compañeros o compañeras.					
2. El profesorado tiene preferencias por ciertos alumnos o ciertas alumnas.					
3. El alumnado habla con malos modales al profesorado.					
4. Algunos alumnos esconden pertenencias o material del profesorado para molestarle deliberadamente.					
5. Hay estudiantes que extienden rumores negativos acerca de compañeros y compañeras.					
6. Hay estudiantes discriminados por otros a causa de sus diferencias culturales, étnicas o religiosas.					
7. Ciertos estudiantes roban objetos o dinero del centro educativo.					
8. El profesorado tiene manía a algunos alumnos o alumnas.					
9. Algunos estudiantes son discriminados por sus compañeros por su nacionalidad.					
10. Los estudiantes hablan mal unos de otros.					
11. Ciertos estudiantes roban objetos o dinero de otros compañeros o compañeras.					
12. Hay estudiantes que se sienten solos o solas en las clases, ignorados y rechazados por sus compañeros/as.					
13. El profesorado ridiculiza al alumnado.					
14. Los estudiantes pegan a compañeros o compañeras dentro del recinto escolar.					
15. El alumnado falta al respeto al profesorado en el aula.					
16. El alumnado dificulta las explicaciones del profesorado hablando durante la clase.					
17. El profesorado ignora a ciertos alumnos o alumnas.					
18. Los estudiantes insultan a profesores o profesoras.					
19. Algunos estudiantes rompen o deterioran a propósito material del centro.					
20. El alumnado insulta a sus compañeros o compañeras.					
21. El alumnado protagoniza peleas dentro del recinto escolar.					
22. Algunos estudiantes protagonizan agresiones físicas en las cercanías del recinto escolar.					
23. Determinados estudiantes son discriminados por sus compañeros o compañeras por sus bajas notas.					
24. El profesorado insulta al alumnado.					
25. El alumnado dificulta las explicaciones del profesor/profesora con su comportamiento durante la clase.					
26. El profesorado no escucha a su alumnado.					
27. Ciertos estudiantes se sienten solos en los recreos, ignorados y rechazados por sus compañeros/as.					
28. Ciertos estudiantes se enfrentan desafiantes al profesorado.					
29. Algunos estudiantes son discriminados por sus compañeros/as por sus buenos resultados académicos.					
30. Los estudiantes amenazan a otros de palabra para meterles miedo u obligarles a hacer cosas.					
31. Ciertos profesores o profesoras intimidan o atemorizan a algún alumno o alumna.					
32. Determinados estudiantes dan collejas o cachetes a sus compañeros o compañeras, bromeando.					
33. Hay estudiantes que son discriminados por su físico por otros compañeros o compañeras.					
34. Hay alumnado que ni trabaja ni deja trabajar al resto.					

Los jóvenes hacen muchas cosas con otros chicos o chicas cada día. Probablemente tú haces algunas cosas más a menudo que otras. Lee cada uno de los siguientes enunciados e indica **el grado en que cada uno de ellos te describe** mediante el número correspondiente.

No me describe nada	No me describe casi nada	Me describe poco	Me describe bastante	Me describe mucho	Me describe totalmente
1	2	3	4	5	6

	1	2	3	4	5	6
1. Cuento chistes y mis compañeros o compañeras de clase se ríen.						
2. Intento que mis compañeros o compañeras hagan las cosas a mi manera cuando trabajamos en una tarea grupal.						
3. Doy la cara por otros chicos o chicas cuando alguien dice algo grosero a sus espaldas.						
4. Olvido devolver cosas que otros chicos o chicas me han prestado.						
5. Hago chistes sobre otros chicos o chicas cuando son torpes en los deportes.						
6. Quedo con otros chicos o chicas para salir.						
7. Ayudo a otros chicos o chicas en sus deberes cuando me piden ayuda.						
8. No hago caso a mis compañeros o compañeras cuando me dicen que deje de hacer lo que estaba haciendo.						
9. Les ofrezco ayuda a mis compañeros o compañeras de clase para hacer sus deberes.						
10. Cuando no me gusta el aspecto que tienen otros chicos o chicas, se lo digo.						
11. Escucho cuando otros chicos o chicas quieren hablar sobre un problema.						
12. Me río de otros chicos o chicas cuando cometen errores.						
13. Empujo a los chicos o chicas que no me gustan (o me caen mal).						
14. Cuando quiero hacer algo, intento persuadir a otros chicos o chicas para que lo hagan, aunque no quieran.						
15. Me aseguro de que todos tengan su turno cuando se hace una actividad de grupo.						
16. Cuando estoy con otros chicos o chicas, solo hablo de aquellos temas que me interesan a mi.						
17. Pido consejos a otros chicos o chicas.						
18. Les digo a otros chicos o chicas que son simpáticos.						
19. No hago caso a otros chicos o chicas cuando no estoy interesado o interesada en lo que están hablando.						
20. Miento para salir de un apuro (aprieto).						
21. Siempre les digo a mis compañeros o compañeras lo que tienen que hacer cuando es necesario hacer algo.						
22. Cuando estoy con mi mejor amigo o amiga paso de otros chicos o chicas.						
23. Coqueteo con el novio o novia de mi amigo o amiga cuando me gusta.						
24. Invento cosas para impresionar a otros chicos o chicas.						
25. Cuando pierdo en un juego les digo a mis compañeros o compañeras de clase que jugaron bien.						
26. Me ofrezco para compartir algo con otros chicos o chicas cuando sé que a ellos les gustaría.						
27. Presto dinero a otros chicos o chicas cuando me lo piden.						
28. Pego a otros chicos o chicas cuando me ponen furioso o furiosa.						
29. Le digo a mis compañeros o compañeras de clase que lo siento cuando sé que he dañado sus sentimientos.						
30. Digo la verdad cuando hago algo malo y otros chicos o chicas son culpados o culpadas por ello.						
31. Hablo más que los demás cuando estoy en grupo de chicos o chicas.						
32. No hago caso de otros compañeros o compañeras cuando me hacen cumplidos.						
33. Tiro cosas cuando estoy enfadado o enfadada.						
34. Ofrezco prestar mi ropa a otros chicos o chicas para ocasiones especiales.						
35. Me muestro agradecido con otros chicos o chicas cuando han hecho algo bueno por mí.						
36. Pongo de mi parte cuando trabajo con un grupo de compañeros o compañeras de clase.						
37. Cuando estoy enfadado o enfadada insulto a mis compañeros o compañeras de clase.						
38. Sé guardar secretos.						
39. Digo a otros chicos o chicas cómo siento las cosas realmente.						
40. Comparto mi comida con compañeros o compañeras de clase cuando me lo piden.						

Esto es todo, ¡Gracias por su participación!
